# Effectiveness of Early Antiretroviral Therapy Initiation to Improve Survival among HIV-Infected Adults with Tuberculosis: A Retrospective Cohort Study

**DOI:** 10.1371/journal.pmed.1001029

**Published:** 2011-05-03

**Authors:** Molly F. Franke, James M. Robins, Jules Mugabo, Felix Kaigamba, Lauren E. Cain, Julia G. Fleming, Megan B. Murray

**Affiliations:** 1 Department of Epidemiology, Harvard School of Public Health, Boston, Massachusetts, United States of America; 2 Partners In Health, Boston, Massachusetts, United States of America; 3 Department of Biostatistics, Harvard School of Public Health, Boston, Massachusetts, United States of America; 4 Center for Treatment and Research on AIDS, Malaria, Tuberculosis and Other Epidemics (TRAC Plus), Rwanda Ministry of Health, Kigali, Rwanda; 5 Ruhengeri Hospital, Rwanda Ministry of Health, Ruhengeri, Rwanda; 6 Division of Global Health Equity, Brigham and Women's Hospital, Boston, Massachusetts, United States of America; McGill University, Canada

## Abstract

**Background:**

Randomized clinical trials examining the optimal time to initiate combination antiretroviral therapy (cART) in HIV-infected adults with sputum smear-positive tuberculosis (TB) disease have demonstrated improved survival among those who initiate cART earlier during TB treatment. Since these trials incorporated rigorous diagnostic criteria, it is unclear whether these results are generalizable to the vast majority of HIV-infected patients with TB, for whom standard diagnostic tools are unavailable. We aimed to examine whether early cART initiation improved survival among HIV-infected adults who were diagnosed with TB in a clinical setting.

**Methods and Findings:**

We retrospectively reviewed charts for 308 HIV-infected adults in Rwanda with a CD4 count≤350 cells/µl and a TB diagnosis. We estimated the effect of cART on survival using marginal structural models and simulated 2-y survival curves for the cohort under different cART strategies:start cART 15, 30, 60, or 180 d after TB treatment or never start cART. We conducted secondary analyses with composite endpoints of (1) death, default, or lost to follow-up and (2) death, hospitalization, or serious opportunistic infection. Early cART initiation led to a survival benefit that was most marked for individuals with low CD4 counts. For individuals with CD4 counts of 50 or 100 cells/µl, cART initiation at day 15 yielded 2-y survival probabilities of 0.82 (95% confidence interval: [0.76, 0.89]) and 0.86 (95% confidence interval: [0.80, 0.92]), respectively. These were significantly higher than the probabilities computed under later start times. Results were similar for the endpoint of death, hospitalization, or serious opportunistic infection. cART initiation at day 15 versus later times was protective against death, default, or loss to follow-up, regardless of CD4 count. As with any observational study, the validity of these findings assumes that biases from residual confounding by unmeasured factors and from model misspecification are small.

**Conclusions:**

Early cART reduced mortality among individuals with low CD4 counts and improved retention in care, regardless of CD4 count.

*Please see later in the article for the Editors' Summary*

## Abstract 2

Molly Franke, Megan Murray, and colleagues report that early cART reduces mortality among HIV-infected adults with tuberculosis and improves retention in care, regardless of CD4 count.

## Editors' Summary

**Background:**

HIV infection has exacerbated the global tuberculosis (TB) epidemic, especially in sub-Saharan Africa, in which in some countries, 70% of people with TB are currently also HIV positive—a condition commonly described as HIV/TB co-infection. The management of patients with HIV/TB co-infection is a major public health concern.

There is relatively little good evidence on the best time to initiate combination antiretroviral therapy (cART) in adults with HIV/TB co-infection. Clinicians sometimes defer cART in individuals initiating TB treatment because of concerns about complications (such as immune reconstitution inflammatory syndrome) and the risk of reduced adherence if patients have to remember to take two sets of pills. However, starting cART later in those patients who are infected with both HIV and TB can result in potentially avoidable deaths during therapy.

**Why Was This Study Done?:**

Several randomized control trials (RCTs) have been carried out, and the results of three of these studies suggest that, among individuals with severe immune suppression, early initiation of cART (two to four weeks after the start of TB treatment) leads to better survival than later ART initiation (two to three months after the start of TB treatment). These results were reported in abstract form, but the full papers have not yet been published. One problem with RCTs is that they are carried out under controlled conditions that might not represent well the conditions in varied settings around the world. Therefore, observational studies that examine how effective a treatment is in routine clinical conditions can provide information that complements that obtained during clinical trials. In this study, the researchers aimed to confirm the results from RCTs among a cohort of adult patients with HIV/TB co-infection in Rwanda, diagnosed under routine program conditions and using routinely collected clinical data. The researchers also wanted to investigate whether early cART initiation reduced the risk of other adverse outcomes, including treatment default and loss to follow-up.

**What Did the Researchers Do and Find?:**

The researchers retrospectively reviewed the charts and other program records of 308 patients with HIV, who had CD4 counts≤350 cells/µl, were aged 15 years or more, had never previously taken cART, and received their first TB treatment at one of five cART sites (two urban, three rural) in Rwanda between January 2004 and February 2007. Using this method, the researchers collected baseline demographic and clinical variables and relevant clinical follow-up data. They then used this data to estimate the effect of cART on survival by using sophisticated statistical models that calculated the effects of initiating cART at 15, 30, 60, or 180 d after the start of TB treatment or not at all.

The researchers then conducted a further analysis to assess combined outcomes of (1) death, default, lost to follow-up, and (2) death, hospitalization due to any cause, or occurrence of severe opportunistic infections, such as Kaposi's sarcoma. The researchers used the resulting multivariable model to estimate survival probabilities for each individual, based on his/her baseline characteristics.

The researchers found that when they set their model to first CD4 cell counts of 50 and 100 cells/µl, and starting cART at day 15, mean survival probabilities at two years were 0.82 and 0.86, respectively, statistically significantly higher than the survival probabilities calculated for each of the other treatment strategies, where cART was started later. They observed a similar pattern for the combined outcome of death, hospitalization, or serious opportunistic infection In addition, two-year outcomes for death or lost to follow-up were also improved with early cART, regardless of CD4 count at treatment initiation.

**What Do These Findings Mean?:**

These findings show that in a real world program setting, starting cART 15 d after the start of TB treatment is more beneficial (measured by differences in survival probabilities) among patients with HIV/TB co-infection who have CD4 cell counts≤100 cells/µl than starting later. Early cART initiation may also increase retention in care for all individuals with CD4 cell counts≤350 cells/µl.

As the outcomes of this modeling study are based on data from a retrospective observational study, the biases associated with use of these data must be carefully addressed. However, the results support the recommendation of cART initiation after 15 d of TB treatment for patients with CD4 cell counts≤100 cells/µl and can be used as an advocacy base for TB treatment to be used as an opportunity to refer and retain HIV-infected individuals in care, regardless of CD4 cell count.

**Additional Information:**

Please access these Web sites via the online version of this summary at http://dx.doi.org/10.1371/journal.pmed.1001029.

- Information is available on HIV/TB co-infection from the World Health Organization, the US Centers for Disease Control and Prevention, and the International AIDS Society

## Introduction

Until recently, there was a paucity of high-quality scientific evidence regarding the optimal time to initiate combination antiretroviral therapy (cART) in adults with HIV and tuberculosis (TB) disease. This uncertainty has posed a major challenge for clinicians, who often defer cART in individuals initiating TB treatment because of concern about immune reconstitution inflammatory syndrome (IRIS) [1]–[5], the possibility of drug interactions [6] and adverse side effects [7], and the risk of reduced adherence due to a higher pill burden among individuals receiving concomitant treatment. Deferral of cART is not without risk: higher mortality was observed among HIV-infected TB patients who received cART either late in the course of TB treatment or not at all [8]–[12]. In recognition of this clinical dilemma, a 2005 World Health Organization (WHO) expert panel identified the assessment of the optimal time to initiate cART as the top research priority related to antiretroviral therapy (ART) for people living with HIV and TB [13].

At least four randomized control trials (RCTs) were initiated to determine whether early versus deferred cART improves survival among TB patients [14]. RCTs are often regarded as the gold standard in clinical research because the intervention is randomly allocated and the potential for confounding by unmeasured factors is minimized [15]. Randomization is especially useful for interventions that are preferentially distributed to the sickest individuals in clinical settings; however, RCTs may be a limited study choice for providing guidance on the timing of cART initiation in co-infected patients. First, RCTs require substantial time and financial resources: to date, only one RCT addressing this clinical research question has published results, though two others have reported results in abstract form. Consistent with observational studies, the SAPiT trial in South Africa found higher mortality among individuals who started cART after, versus during, TB treatment [16]. Data from the CAMELIA trial (presented at the 2010 International AIDS Conference) and the SAPiT and STRIDE trials (presented at the 2011 Conference on Retroviruses and Opportunistic Infections) show that, among adults with severe immune suppression, cART initiation 2–4 wk after TB treatment improved survival or AIDS-free survival relative to initiation 8–12 wk after TB treatment [17]–[19]. Thus, the first RCT data to provide guidance on this topic did not become available for a full five years after the WHO expert panel identified this question as a key research priority.

A second limitation of RCTs for the study of the optimal time for cART initiation is that they measure the efficacy of an intervention under controlled conditions that are often difficult to replicate in clinical settings. For example, three of the RCTs on cART timing, including the SAPiT and CAMELIA trials, required smear or culture confirmation of *Mycobacterium tuberculosis* [14],[16]. Since culture is not available to most of the world's TB patients, and HIV-infected patients are often smear-negative, these inclusion criteria apply to only a minority of co-infected patients at clinical sites, as few as 20%–35% in some settings [20]. Such stringent inclusion criteria limit the generalizability of study findings and may result in discrepancies between the efficacy observed in RCTs and the actual effectiveness observed in a clinical setting.

We used routinely collected clinical data from Rwanda, which can be collected efficiently and inexpensively, to evaluate the effectiveness of early cART initiation among eligible HIV-infected adults treated for TB. We utilized appropriate statistical methods to account for potential biases resulting from the observational nature of the data. Our aim was to confirm results from randomized trials among a cohort of HIV-infected adults who were diagnosed with TB under routine programmatic conditions, rather than by sputum smear or culture. We also explored whether early cART initiation reduced the risk of other adverse outcomes, including default and loss to follow-up.

## Methods

### Ethics Statement

This study was approved by the Partners Human Research Committee and the Rwanda National Ethics Committee.

### Study Setting and Population

We conducted this study among HIV-infected cART-eligible adults≥15 y who had not previously initiated lifelong cART and who received a first TB treatment at one of five cART sites (two urban, three rural) in Rwanda between January 2004 and February 2007. The five health facilities were government-run sites that were receiving financial support from the Global Fund to Fight AIDS, Tuberculosis, and Malaria, and additional financial and implementation support from a non-governmental organization (Partners In Health or Médecins Sans Frontières). Both ambulatory patients and individuals who were interned at the clinic or an on-site hospital were eligible for inclusion. Physicians initiated cART according to the Rwanda Ministry of Health eligibility criteria during the study period (WHO HIV disease stage 4 regardless of CD4 cell count, WHO HIV disease stage 2 or 3 with a CD4 cell count < 350; WHO HIV disease stage 1 with a CD4 cell count < 200) [21]. Because TB disease corresponds to WHO HIV disease stage 3, for this analysis, we considered an individual to be cART eligible if s/he had a documented CD4 cell count≤350 cells/µl prior to, or during, TB treatment. TB regimens were directly observed and consisted of rifampin, isoniazid, pyrazinamide and ethambutol. cART was directly observed at least once per day for individuals who received treatment at the rural sites and was self-administered by individuals treated at the urban sites. The first-line cART regimen for HIV-infected individuals on TB treatment consisted of stavudine or zidovudine, lamivudine and efavirenz. Nevirapine replaced efavirenz in individuals who were not receiving TB treatment. Cotrimoxazole was routinely prescribed to individuals with CD4 cell count < 350 cells/µl or WHO HIV disease stage 2, 3, or 4. CD4 cell counts were conducted prior to cART initiation and every 6 mo thereafter [21].

### Data Collection and Study Design

We conducted a retrospective review of each patient's TB and HIV charts and other program records to collect baseline demographic and clinical variables as well as relevant clinical follow-up data. Follow-up for each person began on the TB treatment initiation date or the date of the first CD4 cell count≤350 cells/µl, whichever came later. Because a CD4 cell count≤350 cells/µl was required for study inclusion and was not documented for some individuals until after TB treatment initiation, we excluded person-time corresponding to the period between TB treatment initiation and first CD4 cell count≤350 cells/µl (a total of 7,130 person-days) because this was “immortal person-time,” e.g., person-time that precedes completion of study entry criteria [15]. Patients had the potential to be followed for at least 1 y and a maximum of 2 y after TB treatment initiation.

### Exposure and Outcome Definitions

Because the therapeutic effect of cART is gradual [22], and deaths immediately following cART initiation are likely due to advanced disease, we incorporated a lag of 15 d before an individual was considered to be “on cART.” We assessed death as the primary outcome, and individuals who defaulted (e.g., stopped treatment without clinician approval), were lost to follow-up, or were followed for less than 2 y were censored on their last day of follow-up. We conducted secondary analyses with composite endpoints of (1) death, default, or lost to follow-up; and (2) death, hospitalization due to any cause, or any of the following WHO HIV disease stage 3 or 4 opportunistic infections: cryptococcal meningitis, esophageal candidiasis, HIV encephalopathy, Kaposi's sarcoma, lymphoma, pneumonia/pneumopathy, or recurrent TB. For this endpoint, we included hospitalizations that began at least 1 wk after TB treatment initiation to avoid those that coincided with the initial TB diagnosis. Individuals were considered lost to follow-up 2 mo after their last visit if they (1) were reported by a clinician to have defaulted or been lost to follow-up, (2) were not documented to have received HIV care after completion of TB treatment, or (3) had no visit notes or laboratory results within 4 mo of the 2-y follow-up period or date of chart review, whichever came first. This third criterion was used to account for the fact that losses to follow-up may go unnoticed by clinicians if the patient does not subsequently return to the center.

### Statistical Analyses

We first estimated the mortality hazard ratios of “on cART” and time “on cART,” using a marginal structural Cox proportional hazards model. To do this, we fit a logistic regression model pooled over time and subject with death at day *t* as the outcome and time-varying variables for “on cART” at day *t*, and number of days “on cART” at day *t*. The following baseline covariates were also included in the model: the value of the first CD4 cell count≤350 cells/µl (henceforth, first CD4 cell count) (linear), an interaction term between first CD4 cell count and “on cART,” age≥43 y (75^th^ percentile), gender, site (rural versus urban), in-patient at a health facility at TB treatment initiation (versus out-patient), lack of a CD4 cell count at the time of TB treatment initiation, time between TB start and first CD4 cell count (if positive), and follow-up day. We also considered the location and type of TB and baseline weight in the lowest gender-specific quartile as potential confounding baseline variables for the effect of “on cART,” but since these variables did not change the effect estimate for “on cART” by more than 10% and did not predict mortality at *p* < 0.05, we excluded them from the final model. We used inverse probability weighting to adjust for time-varying confounding by CD4 cell count and in-patient health facility status and to account for the possibility that sicker individuals may have been more likely to have shorter follow-up for reasons other than death (e.g., losses to follow-up) [23],[24]. Further details are described in Text S1.

For all analyses, the most recent CD4 cell count was carried forward until a new result was received, and follow-up day was modeled as a flexible cubic spline with knots at 60, 180, and 360. Although we tested the variable representing time “on cART” for linearity using a stepwise spline regression model with knots at the same locations [25], the spline term was not selected for the final model at a *p* < 0.05, and we therefore used the continuous linear form. We did not find evidence of statistically significant interaction between “on cART” and any other baseline covariates or follow-up time, nor of a third-order interaction between “on cART,” follow-up time, and time between TB treatment initiation and first CD4 cell count.

In the second set of analyses we compared the causal effect of different cART start times on survival by using the weighted estimates from the final multivariable model to estimate survival probabilities for each individual, based on his/her baseline characteristics. We set the “on cART” and time “on cART” variables to estimate the 2-y mean survival probability that would be expected if everyone in the cohort initiated cART a given number of days after TB treatment. Because TB treatment start may be a more relevant reference time point than time since first CD4 cell count, we set the variables for “missing a CD4 cell count at the time of TB treatment initiation” and “time between TB start and first CD4 cell count” to zero, in order to estimate the causal effect of cART among individuals who have a CD4 cell count at the time of TB treatment initiation. We plotted survival probabilities for five cART initiation strategies:start cART 15 d, 30 d, 60 d, or 180 d after TB treatment start, or never start cART. Because we incorporated a 15-d lag for the “on cART” variable, individuals were assumed to not have experienced any effect of cART until 15 d after cART initiation. For example, for the treatment strategy “start cART 15 d after TB treatment start,” the effect of cART was applied 15 d after cART initiation (30 d after TB treatment start). Standard errors and 95% confidence intervals (CIs) for the simulated survival curves were calculated using a nonparametric unconditional bootstrap [26] (*n = *500 bootstrap samples). We tested differences in 2-y survival probabilities by dividing those differences by the standard error of the difference in 2-y survival probabilities from the bootstrap samples (type 1 error probability  = 0.05 using the usual normal quantiles as cutoff for the statistic).

## Results

### Primary Outcome

**Figure 1:**
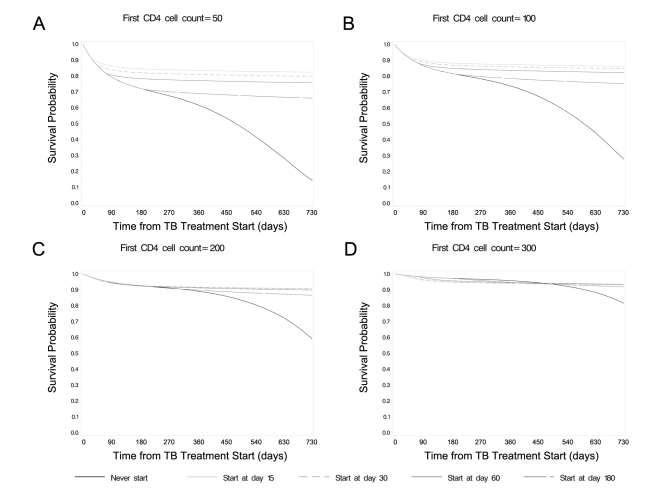
Survival curves for“when to start” strategies, stratified by first CD4 cell count, endpoint of death. (A) Mean probability of survival when first CD4 cell count was set to 50 cells/µl (A), 100 cells/µl (B), 200 cells/µl (C), or 300 cells/µl (D).

Table 1 shows baseline characteristics for the 308 individuals included in the study cohort. Thirty-seven of the 49 deaths during follow-up (75.5%) occurred in the first 6 mo after TB treatment initiation. Time-varying risk factors for cART initiation and censoring are reported in Tables S1 and S2. We found a statistically significant protective effect of cART on mortality, which was greatest among individuals with lower first CD4 cell counts (Table 2; Wald test for interaction, *p*  = 0.03). Figure 1 displays survival probabilities for each of the cART treatment strategies, with first CD4 cell count values set to 50, 100, 200, and 300 cells/µl. When we set first CD4 cell counts to 50 and 100 cells/µl, starting cART at day 15 resulted in mean survival probabilities at 2 y of 0.82 (95% CI: [0.76, 0.89]) and 0.86 (95% CI: [0.80, 0.92]), which were statistically significantly higher than the survival probabilities resulting from each of the other treatment strategies (Figure 1; Table S3). We did not detect statistically significant differences in survival probabilities when we set first CD4 cell counts to 200 or 300 cells/µl, in spite of a tendency toward higher survival probabilities for cART initiation times≤60 d compared to 180 d and never (Table S3). Results were similar when we considered lags of 7 and 21 d before an individual was considered to be “on cART” (results not shown) and when we treated age as a continuous variable.

**Table 1:** Descriptive data for study cohort.

Category	Variable	Binary Variables, Number (%)	Continuous Variables, Median (Range)
**Patient characteristics**	Age (years)		37 (18–77)
	Female gender	187 (60.7)	
**Location and type of TB disease (** ***n = *** **280)** a	Pulmonary, smear positive	47 (16.8)	
	Pulmonary, smear negative	71 (25.4)	
	Pulmonary, smear not done	72 (25.7)	
	Extra-pulmonary, total^b^	90 (32.1)	
	Extra-pulmonary, disseminated	28 (38.4)	
	Extra-pulmonary, ganglion	26 (35.6)	
	Extra-pulmonary, abdominal	11 (15.1)	
	Extra-pulmonary, pleural	7 (9.6)	
	Extra-pulmonary, meningitits	3 (4.1)	
	Extra-pulmonary, pericardial	1 (1.4)	
**CD4 cell count**	CD4 cell count≤350 cells/µl, available at TB start	192 (62.3)	
	If CD4 count not available at TB start, days to first CD4≤350 cells/µl (*n = *116)		45 (2–209)
	First CD4 cell count≤350 cells/µl		113 (1–350)
**cART initiation**	Never started cART	46 (14.9)	
	Time to cART start (days) (*n = *262)		72.5 (0–716)
**In-patient status**	In-patient at health facility at TB start	98 (31.8)	
**Outcome**	Died	49 (15.9)	
	Time to death (days) (*n = *49)		70 (1–669)
	Lost to follow-up or defaulted	32 (10.4)	

*n*  = 308, unless otherwise noted.

a Twenty-eight individuals lacked data on location/type of TB.

b Data on the location of extra-pulmonary TB was available for 73 individuals.

**Table 2:** Multivariable model for the effect of cART and baseline covariates on study outcomes.

Variable	Death (165,164 Person-Days, 49 Events)		Death, Default, Lost to Follow-Up (165,164 Person-Days, 81 Events)		Death, Hospitalization, Serious Opportunistic Infection (140,687 Person-Days, 102 Events)	
	Hazard Ratio [95% CI]	*p*-Value	Hazard Ratio [95% CI]	*p*-Value	Hazard Ratio [95% CI]	*p*-Value
“On cART”	0.3 [0.1, 1.1]	0.06	0.3 [0.1, 0.6]	0.0005	0.4 [0.2, 0.8]	0.02
Weeks “on cART”^a^	1.0 [0.9, 1.0]	0.005	1.0 [0.9, 1.0]	0.009	1.0 [0.9, 1.0]	0.10
First CD4 cell count (per 20-cell increase, linear)	0.8 [0.7, 0.9]	0.002	0.9 [0.9, 1.0]	0.003	0.9 [0.8, 1.0]	<0.001
Interaction between “on cART” and value of first CD4 cell count≤350 cells/µl	1.2 [1.0, 1.4]	0.03	^—b^		1.1 [1.0, 1.2]	0.03
Missing a CD4 cell count at TB treatment start	0.6 [0.3, 1.4]	0.25	1.2 [0.6, 2.2]	0.62	0.9 [0.5, 1.6]	0.65
Weeks between TB treatment start and first CD4 cell count≤350 cells/µl, if positive^a^	1.0 [1.0, 1.1]	0.55	1.0 [1.0, 1.1]	0.57	1.0 [0.9, 1.0]	0.48
Age≥43 y (75^th^ percentile)	1.7 [0.9, 3.4]	0.12	1.1 [0.6, 2.0]	0.69	1.5 [0.9, 2.4]	0.10
Female gender	1.6 [0.8, 3.2]	0.20	1.1 [0.6, 1.8]	0.84	1.3 [0.8, 2.0]	0.32
Rural treatment site	1.4 [0. 7, 3.0]	0.36	0.9 [0.5, 1.6]	0.71	0.8 [0.4, 1.3]	0.32
In-patient at health facility at TB start	2.1 [1.1, 4.3]	0.04	1.4 [0.9, 2.5]	0.17	1.8 [1.1, 2.8]	0.02

Estimates are adjusted for all other variables in model and follow-up day, most recent CD4 cell count, and current hospitalization.

a Days “on cART” and days to first CD4 cell count≤350 cells/µl transformed to the week scale.

b The interaction between “on cART” and value of first CD4 cell count≤350 cells/µl was not statistically significant for the combined endpoint of death, default, and lost to follow-up and was therefore not included in this multivariable model.

### Secondary Outcomes

**Figure 2:**
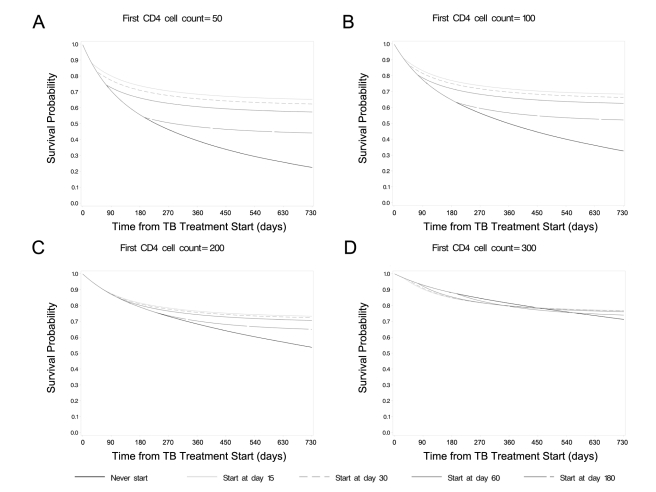
Survival curves for“when to start” strategies, stratified by first CD4 cell count, endpoint of death, serious opportunistic infection, or hospitalization. Mean probability of survival without incident of serious opportunistic infection or hospitalization when first CD4 cell count was set to 50 cells/µl (A), 100 cells/µl (B), 200 cells/µl (C), 300 cells/µl (D).

**Figure 3:**
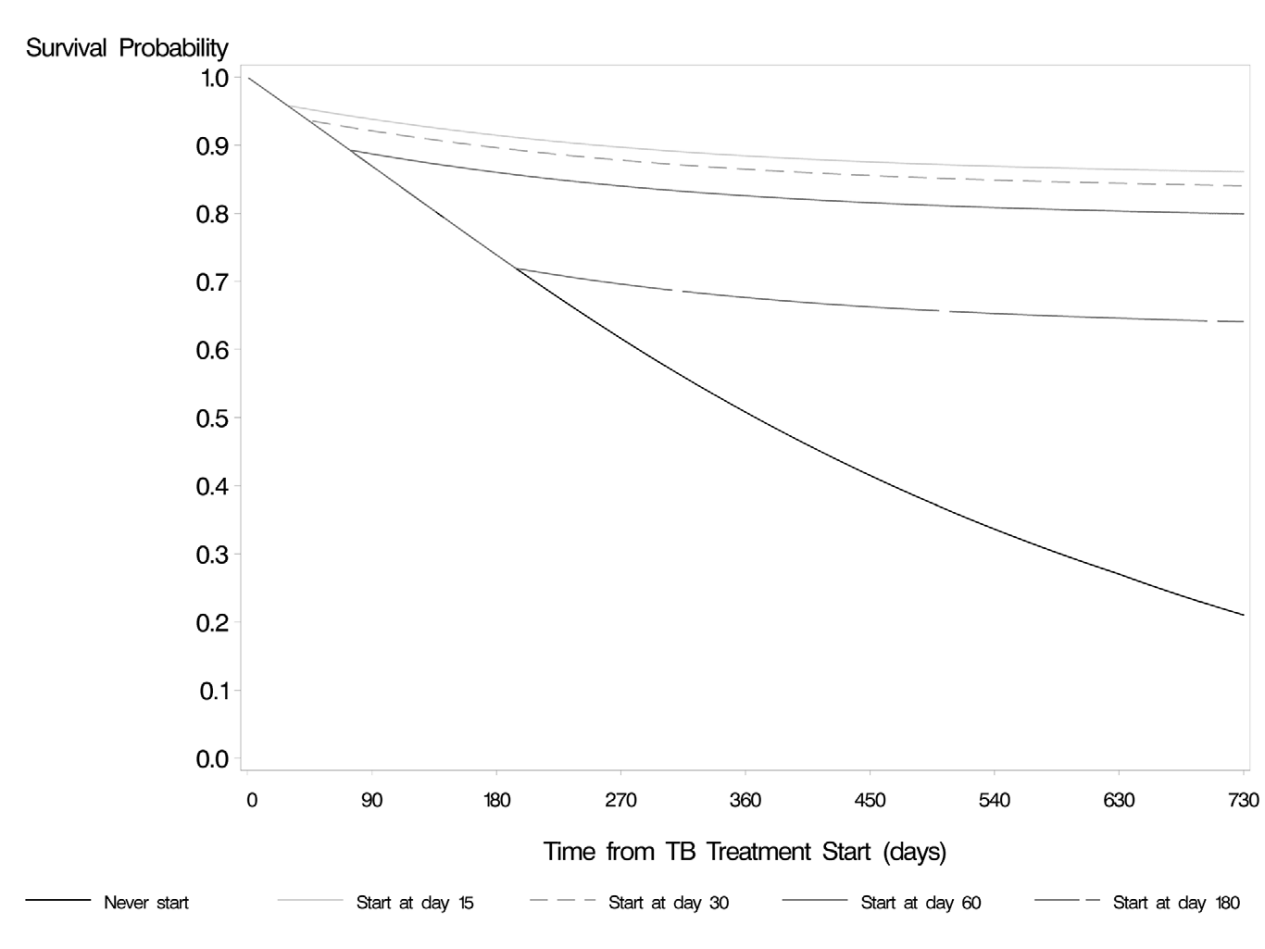
Survival curves for“when to start” strategies, stratified by first CD4 cell count, endpoint of death, lost to follow-up, or default.

The relationship between early cART and the composite endpoint of death, hospitalization, or serious opportunistic infection was similar to that observed for the outcome of death. Initiation of cART 15 d after TB treatment for individuals with CD4 cell counts of 50 or 100 cells/µl yielded a higher probability of remaining free of death, hospitalization, or serious opportunistic infection at 2 y than later initiation of cART (Figure 2; Table S3). We did not observe statistically significant differences in these 2-y probabilities when we set CD4 cell counts to 200 or 300 cells/µl. Compared to later times, cART initiation 15 d after TB treatment was strongly protective against death, default, or loss to follow-up (Figure 3; Table S4), and this effect did not differ by first CD4 cell count.

### Deaths in the First 15 d following cART Initiation

Of the 29 individuals who died after initiation of cART, 11 (37.9%) did so within 15 d of cART initiation. This group demonstrated advanced HIV disease (median CD4 cell count: 54 cells/µl) and initiated cART a median of 53 d after TB treatment (range:8–131 d).

## Discussion

We conclude that cART initiation after 15 d of TB treatment is more beneficial on an absolute scale (measured by differences in survival probabilities) among individuals with TB who have CD4 cell counts≤100 cells/µl, compared with later initiation. Early cART may also improve survival, but less so in terms of absolute risks, for individuals with CD4 cell counts≥200 cells/µl: the difference in survival probabilities was smaller when CD4 cell counts were set to 200 and 300 cells/µl, and we may have lacked statistical power to detect small differences. Given that individuals with CD4 cell counts≥200 cells/µl are eligible for cART in most HIV programs, the higher survival probabilities observed for individuals with a CD4 cell count of 200 cells/µl who initiated cART by day 60, although not statistically significant, suggest that there is no reason to defer cART past 60 d in this group. Early cART may also increase retention in care for all individuals with CD4 cell counts≤350 cells/µl.

Unlike participants in most RCTs, individuals included in this study were diagnosed with TB by a clinician, but were not required to demonstrate sputum that was smear- or culture-positive for *M. tuberculosis*. Because only a small percentage of TB cases in HIV-infected individuals are diagnosed by sputum smear or culture, our results provide evidence of the effectiveness of early cART initiation as it is implemented in most clinical settings in areas with high burdens of HIV and TB. The concept of effectiveness is distinct from, yet complementary to, the efficacy of early cART reported from RCTs [14],[16], which may not be generalizable to the majority of individuals who are treated for TB in the absence of smear or culture confirmation.

The CAMELIA trial was conducted among a group of HIV-infected adults with advanced disease (median CD4 cell count: 25 cells/µl). The authors reported 100-wk survival probabilities of 0.82 (95% CI: [0.78, 0.86]) and 0.73 (95% CI: [0.68, 0.78]) among individuals who initiated cART 2 and 8 wk after TB treatment initiation, respectively [17]. These 2-y survival probabilities are remarkably similar to those we computed for individuals with a first CD4 cell count of 50 cells/µl who initiated treatment 15 d and 60 d after TB treatment initiation: 0.82 (95% CI: [0.76, 0.89]) and 0.76 (95% CI: [0.68, 0.83]), respectively. Our results are also consistent with the SAPiT and STRIDE results, which found that cART initiation after 2–4 wk improved survival or AIDS-free survival compared to initiation at 8–12 wk only among individuals with severe immune suppression (i.e., CD4 cell counts≤50 cells/µl) [18],[19]. Taken together, we conclude that the RCT results may be generalized to the majority of HIV-infected TB patients. These findings also support the notion that observational data, when combined with appropriate analytic methods, should be considered as an ethical, inexpensive, and time-efficient strategy to address urgent clinical questions when RCT results are pending.

Multiple factors contribute to a clinician's ability to initiate cART promptly. Timely HIV diagnosis and referral to HIV services and CD4 cell count enumeration are required for early cART initiation. Therefore, widely available voluntary counseling and testing services, integrated TB and HIV care, and systems that ensure that patients return promptly for appointments will likely facilitate early cART initiation and further reduce mortality among HIV-infected individuals with TB. Early cART was protective against the composite endpoint of death, default, or lost to follow-up, which suggests that TB treatment might offer a brief and valuable window of opportunity for referral and linkage to HIV services, particularly for individuals who have less advanced HIV disease.

Through the use of a marginal structural model we simulated a randomized trial of five cART treatment strategies (start cART 15, 30, 60, or 180 d after TB treatment initiation or never start). Of course, using marginal structural models to simulate an RCT with our data does not have the guarantees of a true RCT. Rather the validity of our study findings depend on several assumptions. First, we assume that the baseline and time-varying confounders for which we adjusted fully account for differences between those who do and do not initiate cART (and were or were not censored). Second, we assume that the model we used to calculate the survival probability for each day of follow-up is correctly specified and predicts survival accurately. Multiple studies have found cART or CD4 cell count to be the strongest determinants of survival among individuals with TB and HIV [8],[27]–[29]; both of these variables were included in our model. For parsimony, we chose to include only statistically significant interactions between “on cART” and the other variables in our model; however, the relatively small size of this cohort and small number of outcomes may have limited our ability to detect and model important interactions between cART and other variables. We incorporated a lag of 15 d before an individual was considered to be “on cART,” since deaths occurring before this time are likely due to advanced HIV disease. If some of the deaths occurring within the first 15 d of cART had been due to cART (e.g., TB IRIS or toxicity), the incorporation of this lag would overestimate the benefit of cART. However, median time between cART initiation and TB IRIS onset or diagnosis ranges from 11 to 46 d [6],[30], and deaths from TB IRIS and toxicity appear to be rare [2],[30]–[32], suggesting that such bias would be minimal.

The health centers included in this study were government health facilities that were also supported by non-governmental organizations that provided additional resources and support to the facility and to patients. These additional resources may have led to an improved capacity of clinic staff or a higher overall level of care relative to centers without these resources. The study was also conducted during a period of HIV treatment scale-up and program strengthening, which continued in the years following the study period. The absolute survival probabilities reported here may be higher than those at centers without non-governmental support, and may have increased in the years since the study period. However, for these factors to limit the generalizability of our finding that earlier cART initiation improves survival relative to later initiation, the effect of cART initiation among cART-eligible patients with TB would need to differ according to the level of health centers' resources or overall quality of care.

In conclusion, we recommend cART initiation after 15 d of TB treatment for those with CD4 cell counts≤100 cells/µl and by day 60 for individuals with CD4 cell counts of 101–200 cells/µl, and advocate for TB treatment to be used as an opportunity to refer and retain HIV-infected individuals in care, regardless of CD4 cell count. We also support prioritization of financial and human resources to maximize the quality and utilization of observational data, which are plentiful in TB and HIV programs throughout the world and may be informative for examining the effectiveness of different treatment strategies. Although the biases associated with use of these data must be carefully addressed, failure to draw upon the experiences of national treatment programs may come at a formidable cost to clinicians, patients, and their families as they await results from RCTs.

## Supporting Information

Table S1 **Time-varying risk factors for cART initiation and censoring in multivariable analysis, primary outcome.**
(DOC)

Table S2 **Time-varying risk factors for cART initiation and censoring in multivariable analysis, secondary outcomes.**
(DOC)

Table S3 **Two-year survival probabilities for different** “**when to start**” **strategies, stratified by first CD4 cell count.**
(DOC)

Table S4 **Two-year probabilities for remaining alive and on treatment for different** “**when to start**” **strategies.**
(DOC)

Text S1 **Inverse probability weighting to adjust for time-varying covariates.**
(DOC)

## Abbreviations

ART: antiretroviral therapy
cART: combination antiretroviral therapy
CI: confidence interval
IRIS: immune reconstitution inflammatory syndrome
RCT: randomized control trial
TB: tuberculosis
WHO: World Health Organization
