# Supplementary material for: Effectiveness of Early Antiretroviral Therapy Initiation to Improve Survival among HIV-Infected Adults with Tuberculosis: A Retrospective Cohort Study
Source: PLoS Med. 2011 May 3;8(5):e1001029. doi: 10.1371/journal.pmed.1001029 (PMC3086874; doi:10.1371/journal.pmed.1001029)
Supplement: Table S1 — Time-varying risk factors for cART initiation and censoring in multivariable analysis, primary outcome. (DOC) [file pmed.1001029.s001.doc]

Table S1. Time Varying Risk Factors for CART initiation and Censoring in Multivariable Analysis, Primary Outcome ^a^

| Variable | cART initiation  27,433 person-days, 262 events | | Censoring ^b^  165,164 person-days, 72 events | |
| --- | --- | --- | --- | --- |
| Most recent CD4 cell count (per 20 cell/μL increase, linear) | 0.9 [0.9, 1.0] | 0.008 | 1.0 [0.9, 1.0] | 0.03 |
| Current in-patient at a health center or hospital | 1.2 [0.8, 1.9] | 0.32 | 0.8 [0.2, 3.4] | 0.76 |
| cART | -- |  | 0.3 [0.1, 0.5] | <0.001 |

a. Estimates are adjusted for follow-up day, site (rural versus urban), gender, age ≥ 43 years, in-patient at health facility at TB start (binary), first CD4 cell count ≤ 350 cells/μL (continuous), no CD4 available at TB start, time between TB treatment start and first CD4 cell count, if positive (continuous).

b. Data are for main analysis with death as the outcome. All other individuals with follow-up time less than two years were censored.
