# Supplementary material for: Effectiveness of Early Antiretroviral Therapy Initiation to Improve Survival among HIV-Infected Adults with Tuberculosis: A Retrospective Cohort Study
Source: PLoS Med. 2011 May 3;8(5):e1001029. doi: 10.1371/journal.pmed.1001029 (PMC3086874; doi:10.1371/journal.pmed.1001029)
Supplement: Table S2 — Time-varying risk factors for cART initiation and censoring in multivariable analysis, secondary outcomes. (DOC) [file pmed.1001029.s002.doc]

Table S2: Time Varying Risk Factors for CART Initiation and Censoring in Multivariable Analysis, Secondary Outcomes ^a^

|  | Death, default, lost-to-follow-up | | Death, hospitalization, serious opportunistic infection | | | |
| --- | --- | --- | --- | --- | --- | --- |
|  | Censoring ^b^  165,164 person-days, 40 events | | cART initiation  25,771 person-days, 251 events | | Censoring ^c^  140,687 person-days, 53 events | |
| variable | hazard ratio  [95% C.I.] | p-value | hazard ratio  [95% C.I.] | p-value | hazard ratio  [95% C.I.] | p-value |
| Most recent CD4 cell count (per 20 cell/μL increase, linear) | 1.0 [0.9, 1.0] | 0.37 | 0.9 [0.9, 1.0] | 0.01 | 1.0 [0.90, 1.00] | 0.04 |
| Current in-patient at a health center or hospital | 0.6 [1.0, 5.0] | 0.67 | 1.2 [0.7, 1.8] | 0.56 | -- | |
| cART | 1.8 [0.5, 6.7] | 0.36 | -- |  | 0.2 [0.1, 0.5] | <0.001 |

1. Estimates are adjusted for follow-up day, site (rural versus urban), gender, age ≥ 43 years, in-patient at health facility at TB start (binary), first CD4 cell count ≤ 350 cells/μL (continuous), no CD4 available at TB start, time between TB treatment start and first CD4 cell count, if positive (continuous).
2. Censored individuals were those who were followed for less than two years for any reason other than death, default, or loss-to-follow-up. Time-varying risk factors for cART initiation are the same as those shown in Table S3.
3. Censored individuals were those who defaulted, were lost-to-follow-up or who were followed for less than two years for any reason other than death, default, or loss-to-follow-up.
