# Supplementary material for: Effectiveness of Early Antiretroviral Therapy Initiation to Improve Survival among HIV-Infected Adults with Tuberculosis: A Retrospective Cohort Study
Source: PLoS Med. 2011 May 3;8(5):e1001029. doi: 10.1371/journal.pmed.1001029 (PMC3086874; doi:10.1371/journal.pmed.1001029)
Supplement: Table S3 — Two-year survival probabilities for different “when to start” strategies, stratified by first CD4 cell count. (DOC) [file pmed.1001029.s003.doc]

**Table S3**. Two-Year Survival Probabilities for Different “When to Start” Strategies, Stratified by First CD4 Cell Count

| Treatment Strategy | First CD4 cell count | | | | |
| --- | --- | --- | --- | --- | --- |
|  | 50 cells/μL  Survival Probability [95% CI] | 100 cells/μL  Survival Probability [95% CI] | 200 cells/μL  Survival Probability [95% CI] | 300 cells/μL  Survival Probability [95% CI] | |
| Death | | | | | |
| Start ART after 15 days of TB treatment | 0.82 [0.76, 0.89] | 0.86 [0.80, 0.92] | 0.91 [0.84, 0.98] | 0.93 [0.84, 1.0] | |
| Start ART after 30 days of TB treatment | 0.80 [0.73, 0.86] ** | 0.85 [0.79, 0.90] * | 0.90 [0.84, 0.97] ^NS^ | 0.93 [0.85, 1.0] ^NS^ |  |
| Start ART after 60 days of TB treatment | 0.76 [0.68, 0.83] ** | 0.82 [0.76, 0.88] * | 0.90 [0.84, 0.96]  ^NS^ | 0.93 [0.86, 1.0] ^NS^ | |
| Start ART after 180 days of TB treatment | 0.66 [0.52, 0.80] * | 0.75 [0.65, 0.85] * | 0.86 [0.79, 0.94]  ^NS^ | 0.92 [0.84, 0.99] ^NS^ | |
| Never start ART | 0.14 [0.0, 0.63] ** | 0.28 [0, 0.81] * | 0.59 [0.11, 1.0] ^NS^ | 0.81 [0.46, 1.0] ^NS^ | |
| Death, serious opportunistic, hospitalization | | | | | |
| Start ART after 15 days of TB treatment | 0.65 [0.56, 0.74] | 0.68 [0.60, 0.77] | 0.73 [0.62, 0.84] | 0.77 [0.61, 0.93] | |
| Start ART after 30 days of TB treatment | 0.62 [0.54, 0.71] ** | 0.66 [0.59, 0.74] * | 0.72 [0.62, 0.82] ^NS^ | 0.77 [0.62, 0.92]  ^NS^ | |
| Start ART after 60 days of TB treatment | 0.57 [0.48, 0.67] ** | 0.63 [0.55, 0.70] * | 0.71 [0.62, 0.80] ^NS^ | 0.76 [0.63, 0.89]  ^NS^ | |
| Start ART after 180 days of TB treatment | 0.44 [0.29, 0.59] ** | 0.52 [0.40, 0.64] * | 0.65 [0.54, 0.76] ^NS^ | 0.74 [0.62, 0.86]  ^NS^ | |
| Never start ART | 0.22 [0, 0.53] * | 0.33 [0.01, 0.65] * | 0.54 [0.23, 0.84] ^NS^ | 0.71 [0.45, 0.97]  ^NS^ | |

** P-value for test comparing survival probability to “Start at day 15” survival probability < 0.01

* P-value for test comparing survival probability to “Start at day 15” survival probability < 0.05

^NS^ P-value for test comparing survival probability to “Start at day 15” survival probability > 0.05
