# Supplementary material for: Effectiveness of Early Antiretroviral Therapy Initiation to Improve Survival among HIV-Infected Adults with Tuberculosis: A Retrospective Cohort Study
Source: PLoS Med. 2011 May 3;8(5):e1001029. doi: 10.1371/journal.pmed.1001029 (PMC3086874; doi:10.1371/journal.pmed.1001029)
Supplement: Table S4 — Two-year probabilities for remaining alive and on treatment for different “when to start” strategies. (DOC) [file pmed.1001029.s004.doc]

Table S4. Two-Year Probabilities for Remaining Alive and on Treatment for Different “When to Start” Strategies

| Treatment Strategy | Survival Probability [95% CI] |
| --- | --- |
| Start ART after 15 days of TB treatment | 0.86 [0.81, 0.91] |
| Start ART after 30 days of TB treatment | 0.84 [0.79, 0.89] ** |
| Start ART after 60 days of TB treatment | 0.80 [0.74, 0.86] ** |
| Start ART after 180 days of TB treatment | 0.64 [0.56, 0.72] ** |
| Never start ART | 0.21 [0.01, 0.41] ** |

** P-value for test comparing survival probability to “Start at day 15” survival probability <0.01
