# Supplementary material for: Effectiveness of Early Antiretroviral Therapy Initiation to Improve Survival among HIV-Infected Adults with Tuberculosis: A Retrospective Cohort Study
Source: PLoS Med. 2011 May 3;8(5):e1001029. doi: 10.1371/journal.pmed.1001029 (PMC3086874; doi:10.1371/journal.pmed.1001029)
Supplement: Text S1 — Inverse probability weighting to adjust for time-varying covariates. (DOC) [file pmed.1001029.s005.doc]

Text S1: Inverse probability weighting to adjust for time-varying covariates

To adjust for time-varying confounding, we weighted each individual for each follow-up day by the inverse of the probability that s/he received his/her observed treatment history, given the observed time-varying covariates. Similarly, we weighted each individual for each follow-up day by the inverse of the probability that s/he experienced his/her observed censoring history, given the observed time-varying covariates. To create the inverse probability of treatment and censoring weights, we fit separate pooled logistic regression models for the probability of remaining off of combination antiretroviral therapy (cART) and for remaining uncensored for each person for each day [1, 2]. Included in these models were the same baseline variables that were included in the model with mortality as the outcome. We also included time-dependent variables for follow-up day, most recent CD4 cell count (linear), and current in-patient at a health facility. A time-varying variable for cART (without the 15 day lag) was also included in the censoring model. Time-varying predictors of cART initiation and censoring are shown in Tables S3 and S4 for primary and secondary outcomes, respectively. We stabilized both the treatment and censoring weights by multiplying them by the probability of remaining off treatment or the probability of remaining uncensored, respectively, given the baseline variables in the model. Use of these weights creates a population in which cART initiation and censoring are not predicted by the time-varying confounders that are included in the models to create the weights [1,2]. The precise mathematical meaning of this somewhat informal statement is discussed in the cited references*.* The means of the stabilized weights were 1.00 [range: 0.35, 4.11], 1.00 [range: 0.12, 2.17], and 1.00 [range: 0.29, 4.93] for outcomes of (1) death; (2) death, default, or lost-to-follow-up; (3) and death, hospitalization and serious opportunistic infections, respectively.

REFERENCES

1. Robins JM, Hernán MA, Brumback B (2000) Marginal structural models and causal inference in epidemiology. Epidemiology 11(5): 550-560.

2. Hernán MA, Hernández-Díaz S, Robins JM (2004) A structural approach to selection bias. Epidemiology 15(5): 615-625.
